# Comparing Life’s Simple 7 and Life’s Essential 8 With Risk of Heart Failure

**DOI:** 10.1016/j.jacadv.2025.102127

**Published:** 2025-09-12

**Authors:** Inge G. van Loon, Yvonne T. van der Schouw, M. Louis Handoko, W.M. Monique Verschuren, Alicia Uijl

**Affiliations:** a Julius Center for Health Sciences and Primary Care, University Medical Center Utrecht, Utrecht, the Netherlands; b Department of Rehabilitation Medicine, Erasmus MC, University Medical Center Rotterdam, Rotterdam, the Netherlands; c Division of Heart and Lungs, Department of Cardiology, University Medical Center Utrecht, Utrecht, the Netherlands; d National Institute for Public Health and the Environment (RIVM), Bilthoven, the Netherlands; e Department of Cardiology, Amsterdam Cardiovascular Sciences, Amsterdam University Medical Center, University of Amsterdam, Amsterdam, the Netherlands; f Division of Cardiology, Department of Medicine, Karolinska Institute, Stockholm, Sweden

**Keywords:** comparison, heart failure, ideal cardiovascular health, Life’s Essential 8, Life’s Simple 7, prospective

## Abstract

**Background:**

Better cardiovascular health (CVH) lowers risk of heart failure (HF). CVH can be quantified using Life’s Essential 8 (LE8), a score consisting of 8 health factors and behaviors.

**Objectives:**

The authors assessed the association between LE8 and risk for HF, and compared LE8 with its predecessor, Life’s Simple 7 (LS7).

**Methods:**

We included 37,803 participants from the EPIC-NL (European Prospective Investigation into Cancer and Nutrition-Netherlands) cohort. The LE8 score ranged from 0 to 100 and was categorized into low (0-49), moderate (50-79), and high (80-100) CVH. CVH classification was compared between LS7 and LE8 scoring. Adjusted Cox proportional hazard models were used to assess LE8 score with risk of HF, and CVH reclassification from LS7 to LE8.

**Results:**

Participants were predominantly female (74%), with a median age of 52 years (Q1-Q3: 42-58). Compared to low CVH (4%), those with high CVH (21%) had an 82% lower risk of developing HF (HR: 0.18; 95% CI: 0.12-0.26). CVH classification differed substantially between LE8 and LS7, with 75% vs 36% having moderate CVH, respectively, which could be attributed to an upward shift (90%) of participants from LS7 low CVH to LE8 moderate CVH. A graded, nonlinear association with risk for HF was observed for LE8, especially for moderate CVH scores.

**Conclusions:**

LS7 and LE8 differ in scoring and CVH classification. While a strong inverse association exists between LE8 score classification and HF risk, the association is graded over the whole LE8 range, suggesting that nuances may be overlooked when using the proposed CVH classification for exploring HF risk.

## Highlights

• Better CVH, either assessed with Life’s Simple 7 or Life’s Essential 8, lowers risk of HF.
• Life’s Essential 8 is inversely and nonlinearly associated with HF risk.
• Life’s Essential 8 differs from Life’s Simple 7 in how participants’ CVH is categorized.
• Future studies should investigate how improvements in CVH scores may lower HF risk.

With 64.3 million people suffering from heart failure (HF) globally, it could be considered a cardiovascular pandemic.^1^^,^^2^ HF is a major public health problem, as it is a complex disease frequently leading to hospitalization or death.^3^ Especially with an expected increase in HF cases due to aging, prevention of this chronic disease is becoming even more important.^4^, ^5^, ^6^ Lifestyle could be a key player in managing cardiovascular health (CVH) and consequently in the prevention of HF.^5^, ^6^, ^7^

To monitor and track CVH, the American Heart Association (AHA) has developed a quantitative scoring system: Life’s Essential 8 (LE8).^8^ LE8 consists of 4 health behaviors (diet, physical activity [PA], nicotine exposure, and sleep health) and 4 health factors (body mass index [BMI], blood lipids, blood glucose, and blood pressure [BP]). LE8 follows after Life’s Simple 7 (LS7) with the addition of a sleep metric and changes in the definition and scoring of metrics, thereby allowing a broader scope of the health behaviors and better capturing interpersonal and intrapersonal differences.^8^^,^^9^ However, only a few recent studies have drawn the comparison between LS7 and LE8, and it is not well-known how the changes in quantifying CVH may affect associations with cardiovascular disease (CVD) risk, and what the added value of the sleep metric is.^10^, ^11^, ^12^, ^13^

Several studies have already shown an inverse association between CVH score and risk of an array of CVD incidences and mortality, including incident HF.^14^, ^15^, ^16^, ^17^, ^18^, ^19^, ^20^, ^21^ However, the majority of these studies investigated LS7.^14^^,^^19^, ^20^, ^21^ Therefore, the aim of the present study is 2-fold: to investigate the association between LE8 score and risk of incident HF and to examine the differences and similarities between LS7 and LE8 scoring methods using the population-based EPIC-NL (European Prospective Investigation into Cancer and Nutrition–Netherlands) cohort.

## Methods

### Study population

EPIC-NL consists of the 2 Dutch contributions to the EPIC cohort. The Prospect-EPIC study includes 17,357 women aged 49 to 70 years at baseline, who participated in the national breast cancer screening program and were living in the city of Utrecht and its surroundings.^22^ The MORGEN (Monitoring Project on Risk Factors for Chronic Diseases)-EPIC cohort consists of a population-based sample of 22,653 men and women aged 20 to 59 years.^23^ The EPIC-NL study was conducted according to the guidelines in the Declaration of Helsinki, and all procedures involving the participants were approved by the Institutional Review Board of the University Medical Center Utrecht (Prospect) and the medical ethical committee of TNO Nutrition and Food Research (MORGEN). Both cohorts were set up between 1993 and 1997 and merged into the EPIC-NL study in 2007 to maintain and expand the cohort data and biobanks.^24^ All participants signed informed consent prior to study inclusion and were asked permission for linkage with disease and mortality registries. We excluded participants that did not consent to linkage with disease or mortality registries (N = 1,304), had prevalent HF (N = 47), missing outcome data (N = 500), or an extreme basal metabolic rate (ratio energy expenditure to energy intake in lowest or upper 0.5%, N = 356) resulting in a final sample for analysis of 37,803 people (Supplemental Figure 1). This study is reported as per the Strengthening the Reporting of Observational Studies in Epidemiology (STROBE) guideline (Supplemental Table 1).

### Data collection and preparation

At baseline, participants filled out a general questionnaire regarding, for example, demographic characteristics, chronic disease history, information on lifestyle, including PA, as well as a validated EPIC food frequency questionnaire (FFQ).^25^ The EPIC FFQ assessed the usual consumption over the past year of 178 main food items, which could be clustered into 20 food groups. PA was assessed in hours per week in the previous year for occupational, recreational, and household activities.^23^ The validated Cambridge Physical Activity Index was used to categorize participants into inactive, moderately inactive, moderately active, or active.^26^ Participants underwent a physical examination, where anthropometric and BP measurements were taken, and a nonfasting 30 mL blood sample was drawn. Bodyweight was measured in light clothing, without shoes, using a floor scale and rounded to the nearest 0.5 kg. BMI was calculated as weight divided by height squared (kg/m^2^).^24^ Systolic and diastolic BP measurements were taken twice in a supine position using a Boso Oscillomat (in Prospect) or using a random zero sphygmomanometer (in MORGEN), and averaged. Hypertension was defined as one or more of the following criteria: systolic BP >140 mm Hg, diastolic BP >90 mm Hg, self-reported use of hypertensive medication, or self-reported hypertension. Total cholesterol was measured using enzymatic methods. High-density lipoprotein (HDL)-cholesterol and low-density lipoprotein-cholesterol were measured using a homogeneous assay with enzymatic endpoint. Glycated hemoglobin was measured in erythrocytes using an immunoturbidimetric latex test.^24^ Questionnaires were sent every 3 to 5 years to assess lifestyle changes, follow-up questionnaires 3 (2011) and 4 (2015) contained a question on sleep duration.

Education was categorized into lower (primary education, advanced elementary education, 3 years of secondary education), middle (completed secondary education, lower or intermediate vocational education), and higher (higher vocational education or university) according to UNESCO (United Nations Educational, Scientific, and Cultural Organization) guidelines.^27^ Extreme values for BMI (≥50 kg/m^2^), sleep duration (<3 h or >16 h),^28^^,^^29^ plasma glucose (≤40 mg/dL or ≥400 mg/dL), and glycated hemoglobin (≤3.29% or ≥13.57%) were set to missing.

### Life’s essential 8

LE8 score consists of the components BMI, blood lipids, blood glucose, BP, diet, PA, nicotine exposure, and sleep health. Each component is scored on a scale of 0 to 100. Definitions of LE8 metrics were based on Lloyd-Jones et al^8^ (Supplemental Table 2). All components were averaged to obtain final LE8 score, thus ranging from 0 to 100. Participants’ scores were categorized into low (0-49), moderate (50-79), or high (80-100) CVH based on the AHA’s proposed cutoffs.^8^

From the PA questionnaire, occupational activity levels, and Metabolic Equivalent of Task (MET)-hours per week of recreational and household activity were obtained. Minutes of moderate to vigorous activity per week were calculated by dividing the MET-hours by the METs for the respective activity and multiplying by 60. Activities with MET ≥4.0 were considered as at least of moderate intensity for participants aged <55 years, and activities with MET ≥3.0 for participants ≥55 years of age, according to Dutch PA guidelines.^30^ Furthermore, those with (heavy) manual jobs were assigned the highest score regardless of other activity (Supplemental Table 2). Diet score was based on the Dietary Approaches to Stop Hypertension diet adherence. Information on Dietary Approaches to Stop Hypertension dietary component consumption was retrieved from the EPIC-FFQ. Sleep duration from follow-up questionnaire 3 was used and when missing or implausible, sleep duration from follow-up questionnaire 4 was taken. If both were available, sleep duration was averaged. Nicotine exposure was based on smoking status and second-hand smoke exposure. Inhaled nicotine-delivery system use was not available in EPIC-NL and thus omitted from the score (Supplemental Table 2). For the blood lipid score, non-HDL cholesterol was calculated from total- and HDL-cholesterol values.

### Life’s simple 7

LS7 consists of BMI, blood lipids, blood glucose, BP, diet, PA, and smoking. Definitions and scoring have been described by the AHA, and by Uijl et al specifically for the EPIC-NL cohort (Supplemental Table 3).^21^^,^^31^ Per component, a score of 0, 1, or 2 can be obtained, signifying low, moderate, or ideal performance. The final LS7 score is the sum of all components and ranges from 0 to 14. Scores were categorized into low (0-8), moderate,^9^^,^^10^ or high^11^, ^12^, ^13^, ^14^ CVH.

For PA, the Cambridge Physical Activity Index was used to assign the score.^26^ The diet metric was based on consumption of healthy diet components, derived from the EPIC-FFQ (Supplemental Table 4).

### Outcome

Participants were followed from date of inclusion until HF diagnosis, death, censor date, or end of the study (January 1, 2011). HF incidence included the first event of HF from hospitalization or death. Information on vital status was obtained via linkage of the EPIC-NL cohort with municipal registries, and cause of death was retrieved from linkage with the national Cause of Death Register, defined by the International Classification of Diseases-10th Revision (codes I50, I11.0, I13.0, and I13.2). Death from HF was defined as HF being the reason for death (primary), or a complication of the cause of death (secondary). Diagnosis of HF hospitalization was defined as HF being the primary reason for hospitalization, or HF being one of ten comorbidities of the primary reason for hospitalization (secondary diagnosis). This information was retrieved from linkage with the Hospital Discharge Register, which was coded according to the International Classification of Diseases-Ninth Revision (codes 428, 402.0-402.9 with fifth-digit 1, 404.0-404.9 with fifth-digit 1 or 3).

### Statistical analysis

R software version 4.1.3 (R foundation for Statistical Computing) was used for all analyses. Baseline characteristics were presented across CVH categories (LE8) in mean ± SD for normally distributed variables, median (IQR [Q1-Q3]) for non-normally distributed variables, or percentages for categorical variables. The *mice* package was used for multiple imputation of missing data. Results were pooled across 10 imputed data sets using Rubin’s rules. Variables used in the imputation are indicated in Table 1. LE8 and LS7 components were averaged from all 10 imputed data sets to obtain 1 component value. Consequently, these average component scores were used to calculate total LS7 and LE8 scores. The percentage of missing data is shown in Supplemental Table 5.

**Table 1:** Baseline Characteristics Stratified for Life’s Essential 8 Score

	Overall (N = 37,803)	Low (LE8 0-49) (n = 1,616, 4.3%)	Moderate (LE8 50-79) (n = 28,186, 74.6%)	High (LE8 80-100) (n = 8,001, 21.1%)
Demographics				
Age^b^	51.5 [42.1, 57.7]	53.6 [49.9, 59.2]	52.1 [43.6, 58.4]	47.9 [33.8, 54.3]
Female^b^	74.4	73.7	74.4	76.1
Marital status^a^^,^^b^				
Single, never married	16.7	8.9	13.9	28.2
Married	0.2	73.2	72.4	61.6
Divorced/separated	7.8	9.8	7.9	7.1
Widowed	5.3	8.1	5.7	3.1
Education^a^^,^^b^				
Low	37.4	55.3	40.1	24.6
Middle	42.4	38.6	43.5	39.3
High	20.2	6.1	16.5	36.1
Lifestyle factors				
Smoking^a^^,^^b^				
Current smoker	38.1	77.7	44.9	7.8
Never smoker	47.9	11.2	40.7	78.9
Current passive smoking^a^^,^^b^	51.0	73.6	55.3	31.1
Pack years^a^^,^^b^	3.0 [0.0, 16.0]	21.3 [10.5, 32.8]	5.4 [0.0, 18.0]	0.0 [0.0, 1.7]
Drinking alcohol^a^^,^^b^				
No, never	7.3	8.6	7.3	7.0
No, quit	1.1	2.7	1.1	0.8
<1 drink/week	29.7	33.8	29.6	29.3
Yes	61.9	54.9	62.0	63.0
DASH-diet adherence^a^^,^^b^	24.0 ± 4.8	20.2 ± 3.8	23.5 ± 4.7	26.8 ± 4.4
Number of healthy diet components consumed^a^^,^^b^	2 [1, 3]	2 [1, 2]	2 [1, 3]	2 [2, 3]
Kcal consumed^a^^,^^b^	2,048 ± 606	2,015 ± 636	2,045 ± 609	2,064 ± 591
Type of work^a^^,^^b^				
Sedentary occupation	23.6	15.9	21.7	32.6
Standing occupation	16.2	10.2	15.8	19.2
Manual work	9.8	4.5	9.5	12.4
Heavy manual work	6.1	6.4	6.6	4.2
Nonworker	44.3	62.9	46.5	31.6
Minutes of moderate to vigorous PA/week^a^^,^^b^	713 [344, 1,539]	624 [109, 1,620]	758 [346, 1,632]	619 [368, 1,160]
Sleep duration (h)^a^^,^^b^	7.1 ± 1.1	6.5 ± 1.8	7.0 ± 1.2	7.3 ± 0.9
Clinical measurements				
BMI (kg/m^2^)^a^^,^^b^	25.2 [22.9, 27.9]	30.9 [28.1, 33.9]	25.7 [23.4, 28.2]	23.1 [21.6, 24.6]
Waist:hip ratio^a^^,^^b^	0.82 ± 0.09	0.90 ± 0.09	0.83 ± 0.09	0.78 ± 0.08
Systolic BP (mm Hg)^a^^,^^b^	126 ± 19	145 ± 21	128 ± 19	115 ± 13
Diastolic BP (mm Hg)^a^^,^^b^	78 ± 11	88 ± 11	79 ± 10	72 ± 8
Pulse (beats/min)^a^^,^^b^	73 ± 11	77 ± 12	74 ± 11	72 ± 10
Total cholesterol (mg/dL)^a^^,^^b^	215 ± 42	249 ± 42	220 ± 41	189 ± 34
HDL cholesterol (mg/dL)^a^^,^^b^	57 ± 16	47 ± 13	55 ± 16	62 ± 17
Non HDL-cholesterol (mg/dL)^a^^,^^b^	158 ± 43	202 ± 42	165 ± 41	127 ± 32
Blood glucose (mg/dL)^a^^,^^b^	90 [83, 101]	101 [92, 122]	92 [83, 101]	86 [79, 95]
HbA1c (%)^a^^,^^b^	5.6 [5.2, 6.2]	6.5 [5.8, 8.0]	5.6 [5.2, 6.2]	5.3 [4.9, 5.5]
Comorbidities and medication				
General health^a^^,^^b^				
Bad-moderate	1.1	4.6	1.1	0.4
Moderate	6.7	15.0	7.0	3.9
Reasonable	26.1	41.2	27.3	18.0
Good	52.8	34.6	52.6	57.7
Excellent	13.3	4.6	12.0	20.0
Medication use				
Cholesterol treatment^b^	3.2	11.2	3.5	0.6
Antihypertensives^b^	10.2	32.6	11.3	1.9
Diabetes treatment^a^^,^^b^	3.0	18.6	2.7	0.3
Prevalent T2DM^a^^,^^b^	2.7	20.0	2.5	0.1
Previous AMI^b^	1.4	5.1	1.5	0.3
Prevalent AF^b^	0.1	0.3	0.1	0.0
Previous stroke^a^^,^^b^	1.2	2.9	1.3	0.5
Prevalent cancer^a^^,^^b^	4.2	6.0	4.3	3.6
Familial history of T2DM^a^^,^^b^	19.6	34.9	20.5	13.7
Life’s Simple 7 and Life’s Essential 8 total and component scores				
LS7 score	8.8 ± 2.1	5.0 ± 1.4	8.4 ± 1.7	11.1 ± 1.2
LS7 smoke (ideal score)^a^^,^^b^	58.5	21.3	52.3	88.0
LS7 BMI (ideal score)^a^^,^^b^	48.4	5.6	41.6	81.0
LS7 diet (ideal score)^a^^,^^b^	2.5	1.2	2.2	3.6
LS7 BP (ideal score)^a^^,^^b^	35.2	2.8	28.4	65.9
LS7 blood glucose (ideal score)^a^^,^^b^	95.9	77.5	96.0	99.2
LS7 blood lipid (ideal score)^a^^,^^b^	36.2	8.4	30.0	63.3
LS7 PA (ideal score)^b^	41.6	26.1	40.4	48.9
LE8 score	69.8 ± 11.6	44.5 ± 4.5	66.9 ± 7.7	85.3 ± 4.4
LE8 smoke score^a^^,^^b^	55 [0, 100]	0 [0, 5]	50 [0, 80]	80 [80, 100]
LE8 BMI score^a^^,^^b^	70 [70, 100]	30 [30, 70]	70 [70, 100]	100 [100, 100]
LE8 diet score^a^^,^^b^	50 [25, 80]	0 [0, 25]	25 [0, 80]	80 [50, 80]
LE8 BP score^a^^,^^b^	50 [50, 100]	30 [5, 50]	50 [50, 100]	100 [75, 100]
LE8 blood glucose score^a^^,^^b^	100 [60, 100]	60 [60, 100]	100 [60, 100]	100 [100, 100]
LE8 blood lipid score^a^^,^^b^	60 [40, 100]	20 [0, 40]	40 [20, 60]	100 [60, 100]
LE8 PA score	100 [100, 100]	100 [90, 100]	100 [100, 100]	100 [100, 100]
LE8 sleep health score^a^^,^^b^	90 [70, 100]	70 [70, 90]	90 [70, 90]	90 [90, 100]

Values shown in %, average ± SD, or median [Q1, Q3].

AF = atrial fibrillation; AMI = acute myocardial infarction; BMI = body mass index; BP = blood pressure; DASH = Dietary Approaches to Stop Hypertension; HbA1c = glycated hemoglobin; HDL = high density lipoprotein; LE8 = Life’s Essential 8; LS7 = Life’s Simple 7; PA = physical activity; T2DM = type 2 diabetes mellitus.

a Variable has been imputed;

b Variable has been used for imputation of other variables. Life’s Simple 7 and Life’s Essential 8 components are calculated from the averages of 10 imputed data sets.

Kaplan-Meier curves were created to visualize time to HF incidence. Multivariable Cox proportional hazards models were used to estimate the HRs and 95% CIs for the association of LE8 score (categorical) with incident HF, using the “low” category as reference. Other competing events, such as death due to other CVD or alternative causes, were treated as censored at the time of death. Cohort was added as a strata in the model to account for baseline hazard differences between MORGEN and Prospect. The proportional hazards assumption was verified by assessing the Schoenfeld residuals. Two multivariable models were created. Model 1 was adjusted for sex, age, and education, model 2 was additionally adjusted for marital status, previous acute myocardial infarction, and prevalent atrial fibrillation. Presumed associations between the exposure, outcome, and covariates have been illustrated in a direct acyclic graph (Supplemental Figure 2). We evaluated effect modifications by age and sex by modeling an interaction term with LE8.

For analyzing LE8 score continuously, restricted cubic spline terms were integrated in the LE8 Cox model. The same multivariable adjustments were used as in the categorical analyses. Reference score was set at 50, as this was the cutoff between the low and moderate CVH categories. Analysis was performed for the 10 separate imputed data sets, and the splines were overlaid. *P* value for nonlinearity was obtained.

To investigate the influence of each metric on the association with incident HF, a leave-one-out analysis was performed for each component of the scores, within the categorical LE8 Cox model. A forest plot was created to visualize the results.

Reclassification from LS7 to LE8-determined CVH category was calculated as N (%) of the LS7 CVH category. This reclassification was visualized in a Sankey diagram. Weighted Cohen’s kappa statistic was calculated to assess agreement in categorization.

Based on LS7 and LE8 CVH attribution, participants were categorized according to their LS7 to LE8 CVH reclassification. Reclassification from LS7 low to LE8 high, and LS7 moderate to LE8 high occurred rarely (N = 86 and N = 12, respectively). To prevent issues with model convergence, these participants were included in the most similar categories, with low-high being classified as low-moderate, and moderate-low as moderate-moderate.

To investigate smaller subgroups and the difference between LS7 and LE8 score systems, the association of CVH reclassified groups with risk of HF was analyzed using Cox proportional hazard analysis. Models were adjusted for the same confounders as before.

Sensitivity analyses for the LE8 Cox proportional hazard models were performed where participants with incident HF in the first 2 years of follow-up were excluded to investigate possible reverse-causation bias (N = 35).

## Results

### Baseline characteristics

Overall, the study population included 37,803 participants. Mean LE8 score was 69.8 ± 11.6. Of all participants, 4.3% had low CVH, 74.6% had moderate CVH, and 21.1% had high CVH. Median age was 51.5 years (Q1-Q3: 42.1-57.7) and the majority of the participants were female (74.4%). Those with high CVH (LE8) generally were younger, had higher education, a sedentary occupation, smoked less, and used fewer medications compared to the participants with low or moderate CVH (Table 1).

### Risk for developing heart failure

**Figure 1:**
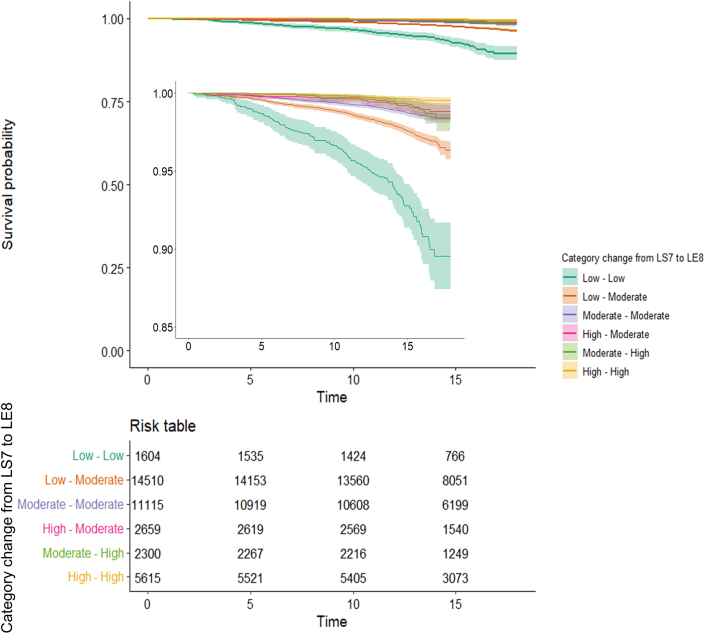
**Kaplan-Meier Curve and Risk Table Stratified for CVH Reclassification From LS7 to LE8** Y-axis depicts heart failure–free survival probability, the x-axis shows time in years. The curves differed significantly (log-rank *P* < 0.001). Inset depicts the zoomed-in curves with y-axis 0.85 to 1.00. LE8 = Life’s Essential 8; CVH = cardiovascular health; LS7 = Life’s Simple 7.

Median follow-up time was 15.3 years (Q1-Q3: 14.1-16.5, max 18.0 years), in which a total of 690 participants (1.8%) developed HF. All Kaplan-Meier curves differed significantly between LE8 and reclassified CVH categories, respectively (log-rank *P* < 0.001) (Supplemental Figure 3, Figure 1).

After multivariable adjustment, those in the high LE8 CVH category had 82% lower risk of developing HF (HR: 0.18; 95% CI: 0.12-0.26), and those in the moderate LE8 CVH category had a 65% lower risk of developing HF (HR; 0.35; 95% CI: 0.26-0.47) compared to the low LE8 CVH group (Table 2). We did not observe effect modifications by age or sex.

**Table 2:** Associations Between Life’s Essential 8 and Reclassified CVH Groups With Risk of HF

CVH Classification	Person-Years at Risk	N_events_/N_Total_	Model 1	Model 2	Model 3
LE8 CVH category					
Low (0-49)	22,716	117/1,616	1.00	1.00	1.00
Moderate (50-79)	418,248	530/28,186	0.29 (0.21-0.39)	0.34 (0.25-0.45)	0.35 (0.26-0.47)
High (80-100)	120,000	43/8,001	0.10 (0.07-0.14)	0.16 (0.11-0.24)	0.18 (0.12-0.26)

LS7 CVH	LE8 CVH					
Categorization changes from LS7 to LE8 scoring system						
Low	Low	22,543	117/1,604	1.00	1.00	1.00
Low	Moderate	213,524	364/14,510	0.37 (0.27-0.51)	0.38 (0.28-0.52)	0.40 (0.30-0.54)
Moderate	Moderate	166,048	142/11,115	0.19 (0.14-0.26)	0.25 (0.19-0.34)	0.27 (0.16-0.43)
High	Moderate	40,132	24/2,659	0.14 (0.09-0.23)	0.25 (0.15-0.41)	0.25 (0.16-0.43)
Moderate	High	34,539	21/2,300	0.14 (0.08-0.24)	0.18 (0.11-0.32)	0.20 (0.11-0.54)
High	High	84,177	2/5,615	0.07 (0.04-0.11)	0.14 (0.08-0.22)	0.14 (0.09-0.24)

Values shown are HR (95% CI). Model 1: crude model; Model 2: adjusted for sex, age, and education; Model 3: adjustments model 2 with addition of marital status, previous acute myocardial infarction, and prevalent atrial fibrillation.

CVH = cardiovascular health; HF = heart failure; N = number; other abbreviations as in Table 1.

In the fully adjusted models, no difference in risk of HF was seen for any of the scores where one metric was left out compared to the complete LE8 scores (Supplemental Figure 4).

**Figure 2:**
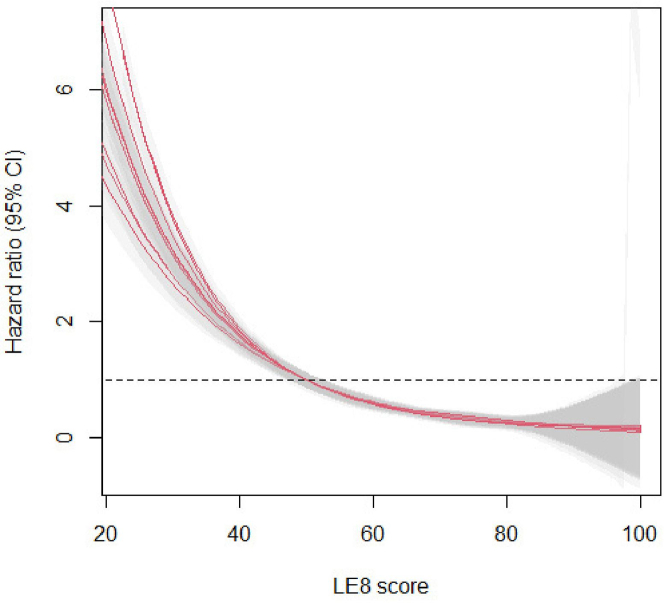
**Restricted Cubic Spline Analysis for LE8 Score and Risk of HF** Shown with 95% CI (shaded gray area). Reference set at LE8 score of 50, dashed line indicates HR = 1. Graphs of 10 imputed data sets are overlaid. HF = heart failure; other abbreviation as in Figure 1.

The restricted cubic spline analysis of the continuous score variable showed a nonlinear association, with a steep decrease in HF risk within the range of low LE8 scores, that gradually flattens out across the moderate and high CVH score range (*P*_non-linearity_ = 0.01) (Figure 2).

Exclusion of HF cases within the first 2 years of follow-up (N = 35) did not affect estimates (Supplemental Table 6).

### Differences in CVH classification between LE8 and LS7

Where 42.6% of participants had low CVH according to LS7, this decreased to 4.3% when using the LE8 score classification (Table 3, Central Illustration). An upward classification of 89.5% of the LS7 low CVH group to the LE8 moderate CVH group was observed. Consequently, the majority of the population classified as having moderate CVH under LE8 (74.6%). Approximately equal sized groups interchanged between the moderate and high CVH categories of LS7 and LE8. Cohen’s weighted kappa was 0.462 (95% CI: 0.456-0.467), indicating moderate agreement between the CVH classification based on LS7 and LE8 scores.

**Table 3:** Overlap in CVH Categorization for LS7 and LE8 Scoring

Life’s Simple 7 Categorization	Life’s Essential 8 Categorization			Total
	Low CVH (n = 1,616, 4.3%)	Moderate CVH (n = 28,186, 74.6%)	High CVH (n = 8,001,21.1%)	
Low CVH (n = 16,114, 42.6%)	1,604 (10.0)	14,424 (89.5)	86 (0.5)	16,114 (100)
Moderate CVH (n = 13,415, 35.5%)	12 (0.1)	11,103 (82.8)	2,300 (17.1)	13,415 (100)
High CVH (n = 8,274, 21.9%)	0 (0.0)	2,659 (32.1)	5,615 (67.9)	8,274 (100)

Values are n (%) reflecting proportion that the respective LE8 category makes up of the original LS7 categorization.

Abbreviations as in Tables 1 and 2.

**Central Illustration:**
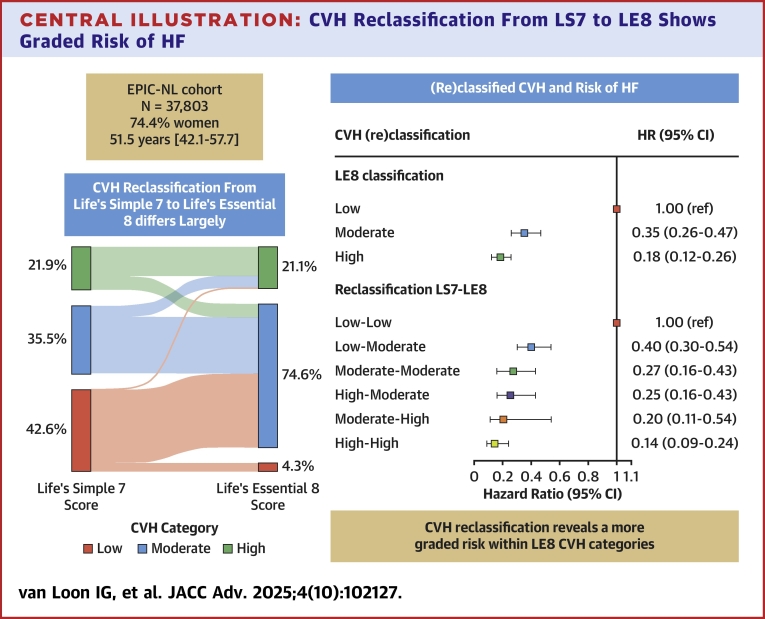
**CVH Reclassification From LS7 to LE8 Shows Graded Risk of HF** Sankey diagram (left) showing the movement of participants from LS7 CVH classification (left) to LE8 CVH classification (right). Right side shows forest plot of the association between (re)classified CVH and risk of heart failure. EPIC-NL = European Prospective Investigation into Cancer and Nutrition–Netherlands; other abbreviations as in Figures 1 and 2.

Within the LE8 moderate CVH category, a graded inverse association with HF risk was seen, with a 60% lower risk of developing HF for those who were upwardly reclassified from LE7 low CVH to LE8 moderate CVH (HR: 0.40; 95% CI: 0.30-0.54), compared to a 75% lower risk for participants who were downwardly reclassified from LE7 high CVH to LE8 moderate CVH (HR: 0.25; 95% CI: 0.16-0.43) (Central Illustration, Table 2). The graded inverse association is also reflected in the Kaplan-Meier curve of the reclassification model, where a clear difference in heart failure–free survival probability between, for example, the low-low and low-moderate groups is visible (Figure 1). For participants reclassified to lower CVH categories, it could be seen that participants, for example, smoked more often, had higher BMI, BP, and cholesterol than those reclassified toward higher LE8 CVH categories (Supplemental Table 7).

## Discussion

Within the EPIC-NL population, 21.1% had high CVH, 74.6% had moderate CVH, and 4.3% had low CVH, according to LE8 scoring. The update from the AHA’s LS7 to LE8 resulted in major changes, causing a large upward shift in participants’ CVH classification. High LE8 CVH is associated with an 82% lower risk of developing HF. The association of LE8 score with HF was graded over the entire score range, with pronounced differences in risk within the LE8 moderate CVH group, depending on reclassification from LS7 to LE8 CVH.

### LE8 score and risk of HF

The EPIC-NL population had similar LE8 scores compared to several other populations, which also showed the largest proportion of participants being classified as having moderate CVH, with percentages ranging between 62.1% and 81.4%.^11^^,^^13^^,^^17^^,^^18^^,^^32^ The clear reduction in risk of HF for higher LE8 scores, and thus better CVH, is in line with results from recent studies in populations with Asian ancestry,^16^^,^^18^ European ancestry,^14^^,^^17^^,^^33^ and an American population.^15^^,^^32^ Some of the studies also reported that the association was graded, similar to our results, with a larger lowering of HF risk for improvements in the lower range of LE8 scores than for higher scores.^14^^,^^17^

### CVH reclassification from LS7 to LE8

We observed a large upward shift in CVH classification when comparing LS7 and LE8 scoring, with almost all participants with LS7 low CVH being reclassified as having moderate CVH under LE8. This large change in CVH classification in line with findings from 2 other studies investigating cross-classification between LS7 and LE8 categories.^11^^,^^13^ However, both studies mostly observed downward CVH reclassification, whereas we noted an upward shift from low to moderate CVH. The use of lower thresholds for LS7 CVH categories (low: 0-4; moderate: 5-9; high: 10-14) in the other studies may explain the downward reclassification, while stricter cutoffs were used in this study. The reclassification to moderate CVH can possibly be explained by the LE8 score system seeming to be more lenient in its CVH classification thresholds than the LS7 system. However, it must be noted that CVH cutoffs for LS7 were determined per study, and not standardized by the AHA. Another reason for the large upward classification could be that almost all participants scored in the high range for the sleep metric (70.3%) and PA metric (96.1%), further increasing average LE8 scores.

### Comparing LS7 to LE8

In our study population, the protective effect of higher LS7 CVH^21^ is less strong than the protective effect of higher LE8. Compared to low LS7 CVH, those in the LE8 low CVH category have worse lifestyle habits, as well as cardiometabolic factors, such as BP or cholesterol. Therefore, it is likely that only the unhealthiest of the LS7 low category remained classified as low in LE8.

A graded, inverse association of the LE8 score with developing HF was observed. This variability was also reported in a previous study. Compared to the reference score of 74, individuals with a score of 60 had a HR: 1.74 (95% CI: 1.68-1.80), and those with a score of 80 had HR: 0.81 (95% CI: 0.78-0.84).^14^ The graded association in our study became more tangible when further separating the large, heterogeneous moderate LE8 CVH group according to CVH reclassification. The highest risk of HF was observed in previously low LS7, compared to lowest risk of HF in previously high LS7. Furthermore, the graded risk was reflected in the clear difference in survival probability for the low-low and the low-moderate group. This also suggests that LE8 may have a better predictive value for HF than LS7 scores.

Apart from the classification shifts, one of the most notable differences between LS7 and LE8 was the addition of the sleep health metric. However, exclusion of the sleep metric in the LE8 score did not affect HRs, replicating a previous study, indicating that sleep was not a very influential metric.^32^ Studies investigating the sleep metric separately did not find a significant association with risk of CVD mortality or HF either.^16^^,^^18^ Addition of sleep was well supported in the LS7 context, as it was associated with each separate metric, and inclusion of a sleep metric in LS7 score improved predictive value for CVD events.^8^^,^^34^ However, it is unclear yet what role it has within LE8. One study suggests that the LE8 scoring method, that is, 0 to 100, rather than addition of sleep, affected associations with outcomes more. Namely, their findings using the LE8 metrics, but with an LS7-type scoring of 0, 1, or 2 points per metric are in line with our findings for LS7 models.^16^^,^^21^ To clarify the added value of the sleep metric within the LE8 context, it is recommended that future studies further investigate this metric, or whether sleep health measures other than self-reported duration may have more added value.

### Clinical relevance and implications

With approximately 80% of the population at nonideal CVH and strong associations with the risk of HF, this study emphasizes the importance of obtaining high CVH.^8^^,^^35^ Improving CVH on population level is crucial, with one study stating that 34% of HF cases could be attributed to low-moderate CVH scores.^17^ Especially as HF develops over several (pre-)clinical stages, LE8 may serve as an early signaling method to identify higher-risk individuals for HF and provide insights in the health factors and behaviors to be improved. Consequently, intervening at an early stage to help individuals improve their CVH can contribute to preventing morbidity and mortality.^7^ This is supported by evidence showing that improvement of CVH score over time lowers the risk of CVD.^36^^,^^37^ While these results are promising, interventions demonstrating that LS7 score improvement is associated with actual risk reduction have not been performed. For LE8 score improvements and CVD risk studies are, to our knowledge, lacking altogether. Future studies could therefore investigate the effects of improvements in LE8 score over time and especially what magnitude of improvement already yields beneficial risk reduction.

Furthermore, the heterogeneity and size of the LE8 moderate CVH group raises the question whether the AHA’s proposed thresholds for CVH classification are useful when wanting to, for example, assess the effects of improving score on individual or population level. Currently, improvements in score for those in the lower part of the moderate category are likely to go unnoticed as people do not change CVH categories. By investigating LE8 score on a continuous scale or into smaller predefined sections, more insight could be gained into the risk reductions that could be obtained even with small changes.

Lastly, goals for score improvement may vary for populations. For example, our Dutch population scored very high on the PA and sleep metric, especially when compared with the guideline adherence in the U.S. population.^38^, ^39^, ^40^, ^41^ Therefore, the focus for population-wide approaches should consider where the population is currently lacking, and not simply aim to improve all metrics. Moreover, LE8 components may not equally contribute to cardiovascular risk, as suggested previously in the ARIC (Atherosclerosis Risk in Communities) study, where almost a quarter of HF cases in the population were attributable to uncontrolled glycemia, BP, and/or obesity. This underlines that it should be investigated for a specific population where maximum health gain can be achieved.

### Strengths and limitations

This study is the first to investigate the influence of CVH reclassification from LS7 to LE8 on HF incidence. This study benefited from the high number of participants, long follow-up, and large availability of information regarding demographics, lifestyle, and disease history. However, information on sleep was not collected at baseline but at later follow-up, unlike the other variables used for LE8 score calculation. Moreover, sleep duration and PA, which formed the basis for 2 of the LE8 components, were self-reported data. Therefore, bias may be present due to underreporting or overreporting. This may have also contributed to the high scores obtained by our population for the LE8 sleep and PA components. Objectively assessed data would be preferred. Additionally, no information on nicotine delivery system use was collected, which forced us to modify calculation of the nicotine exposure metric. However, since electronic nicotine delivery systems had not been developed yet in the data collection period (1993-1997), the risk of misclassification is nonexistent.^42^ Furthermore, the sample was largely of European ancestry (>95%), as shown by previous genetic analysis.^43^^,^^44^ This may affect generalizability to non-European populations.

## Conclusions

To conclude, the LS7 and LE8 scoring systems vary not just in the addition of a sleep metric, but mostly in their scoring methods. Individuals’ CVH classification varies between the systems, and a strong, graded, inverse association with HF risk is seen within LE8 categories. With this range in risk not being fully reflected in the current CVH classification, it is recommended that future research takes a more nuanced approach by, for example, assessing risk continuously or in smaller categories, adapting more to the wide range in health of the population.

Perspectives **COMPETENCY IN MEDICAL KNOWLEDGE:** Individuals with lower CVH have a higher risk of developing HF. However, current CVH classification does not accurately reflect the range in risk.

**TRANSLATIONAL OUTLOOK:** These data underline the benefits of improving CVH. The LE8 scoring system may provide a tool, for example, for general practitioners to screen for risk of CVD, or HF specifically. It also provides an overview for which health factors and behaviors the participant can improve most, providing possibility for a tailored approach. Future research should investigate what magnitude of LE8 score improvement is necessary for health benefits.

## Funding support and author disclosures

Dr Handoko is supported by the 10.13039/100018890Dutch CardioVascular Alliance (2020B008 RECONNEXT) and the Dutch Heart Foundation (NHS; 2020T058). Dr Uijl is supported by the 10.13039/100018890Dutch CardioVascular Alliance, Dutch Heart Foundation, and ZonMw (01-001-2021-B015 HEART4DATA). Dr Handoko has received educational/speaker/consultancy fees from Novartis, Boehringer Ingelheim, Daiichi Sankyo, Vifor Pharma, AstraZeneca, Bayer, MSD, and Quin; all not related to this work. All other authors have reported that they have no relationships relevant to the contents of this paper to disclose.
