# Supplementary material for: Comparing Life’s Simple 7 and Life’s Essential 8 With Risk of Heart Failure
Source: JACC Adv. 2025 Sep 12;4(10):102127. doi: 10.1016/j.jacadv.2025.102127 (PMC12791877; doi:10.1016/j.jacadv.2025.102127)
Supplement: Supplemental Data [file mmc1.pdf]

Supplemental Appendix

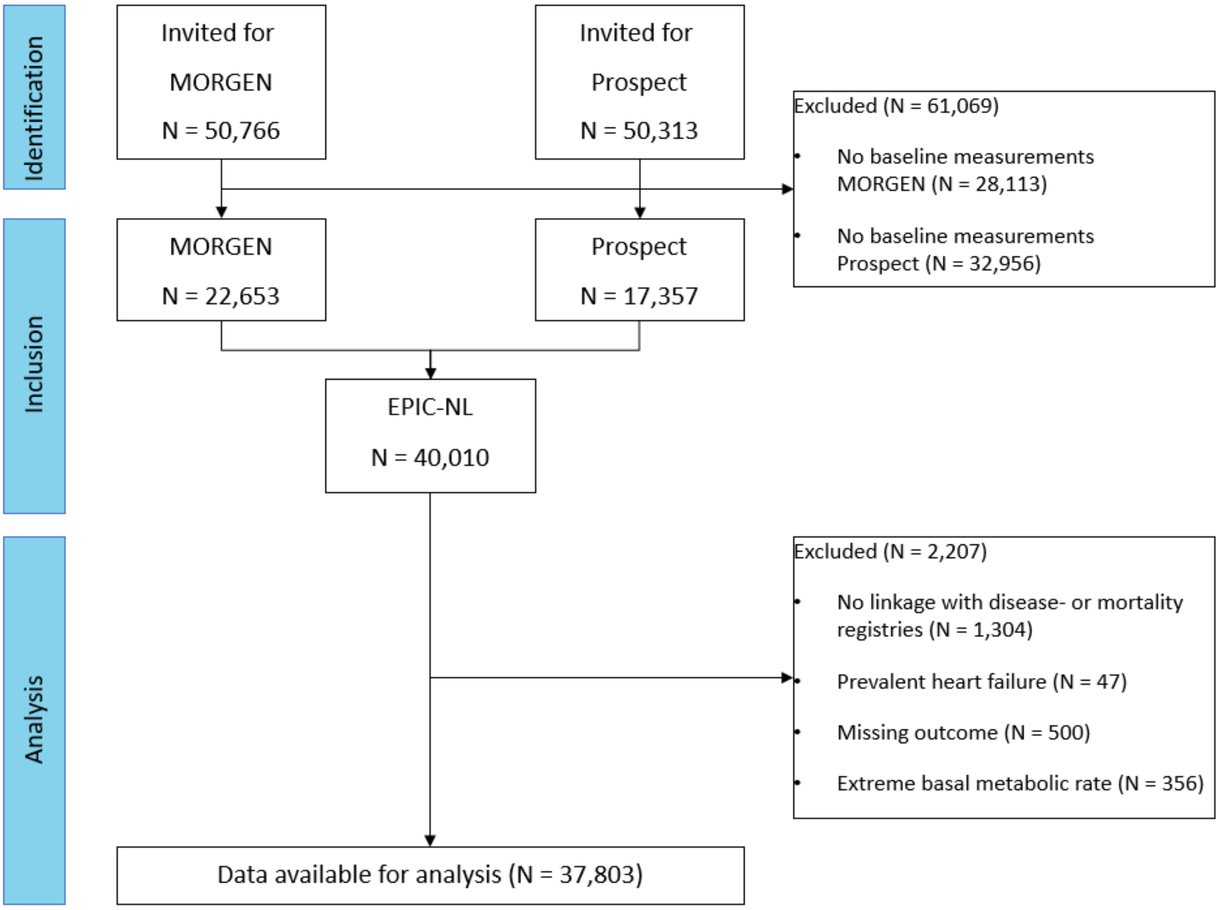

Supplemental Figure 1: Flow chart of participant in- and exclusion

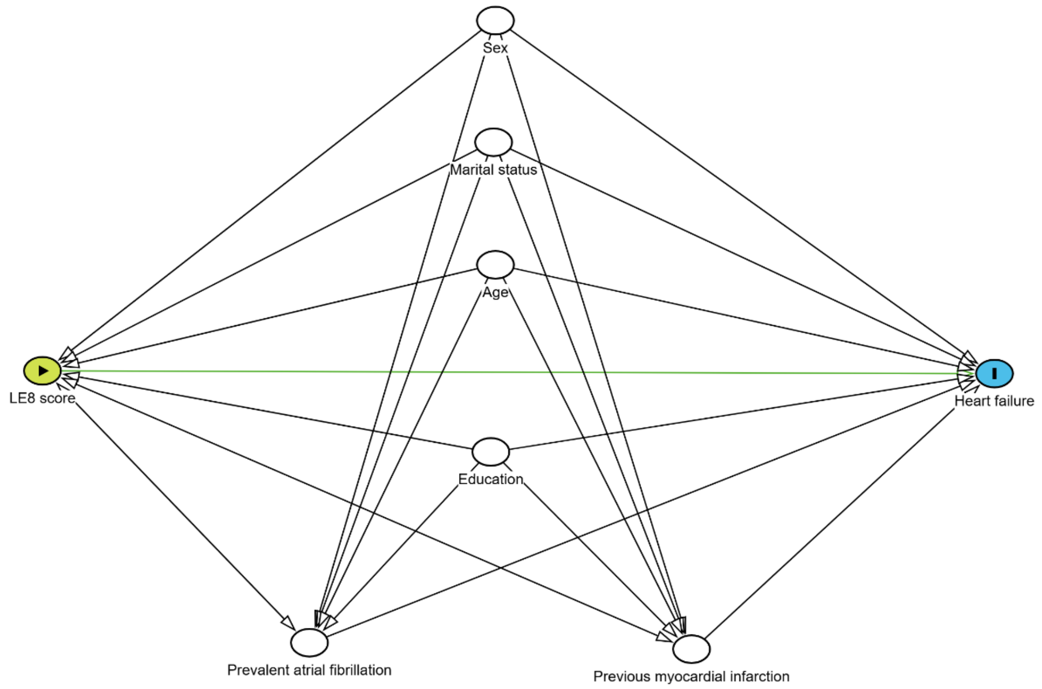

Supplemental Figure 2: Direct Acyclic Graph (DAG) of the presumed associations between exposure (LE8 score), outcome (Heart failure), and covariates with minimally sufficient adjustment.

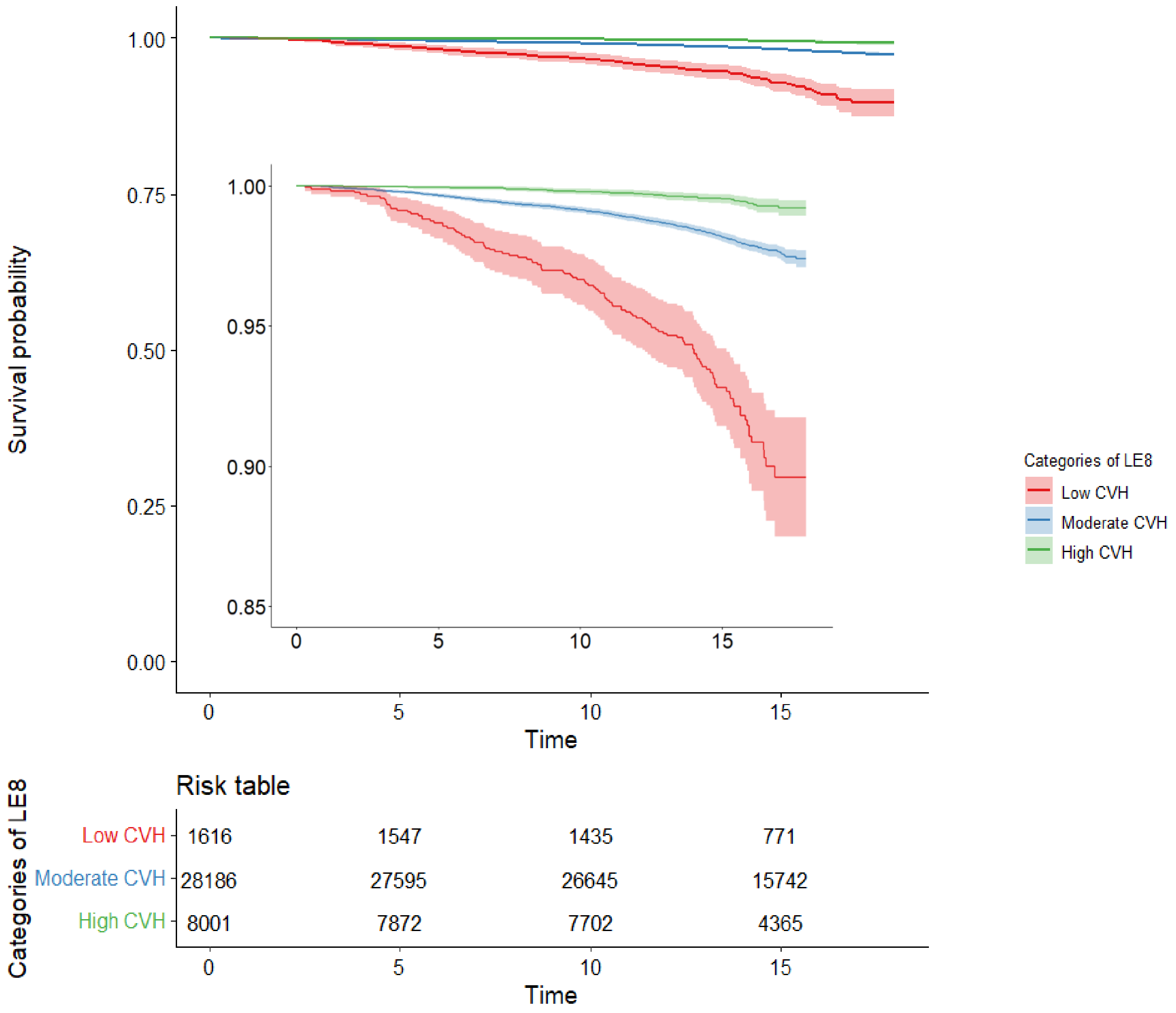

Supplemental Figure 3: Kaplan-Meier curve and risk table stratified for Life's Essential 8 category. The curves for different CVH categories differed significantly (log-rank p<0.001).). Inset depicts the zoomed-in curves with y-axis 0.85 -1.00. CVH = cardiovascular health; LE8 = Life's Essential 8

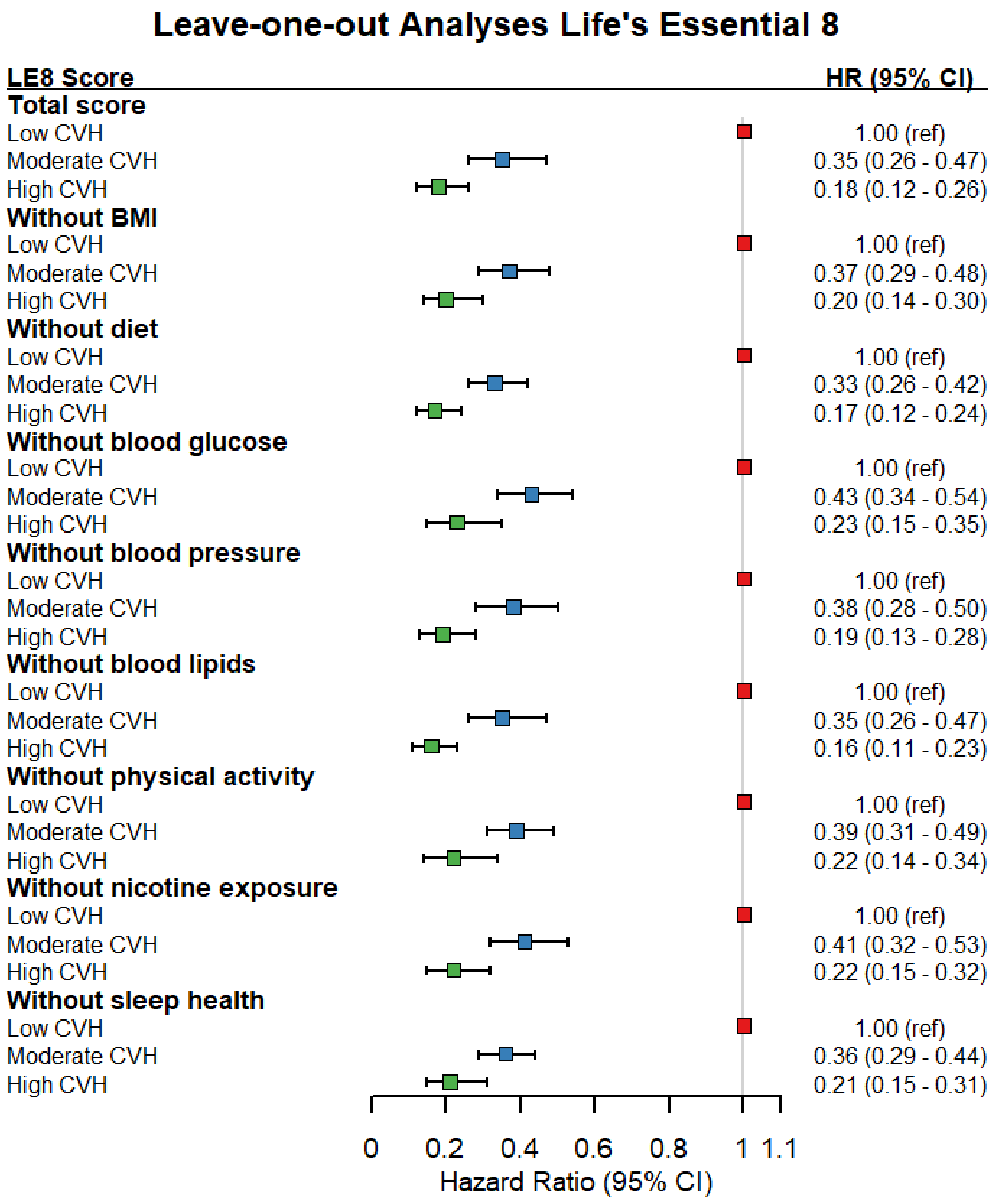

Supplemental Figure 4: Forest plot of HRs from leave-one-out analyses.

A new LE8 score is created by one-by-one leaving out each component to investigate the impact of excluding a component. Models are adjusted for sex, age, education level, marital status, acute myocardial infarction, and atrial fibrillation. 95% CI = 95% confidence interval; BMI = body mass index; HR = hazard ratio; LE8 = Life’s essential 8; ref = reference

Supplemental Table 1: STROBE Statement—checklist of items that should be included in reports of observational studies

	Item No.	*Recommendation*	*Page No.*	*Relevant text from manuscript*
**Title and abstract**	*1*	*(a) Indicate the study's design with a commonly used term in the title or the abstract*	p.3	[We included… EPIC-NL cohort]
		(b) Provide in the abstract an informative and balanced summary of what was done and what was found	p. 3	Abstract section
**Introduction**				
Background/rationale	*2*	*Explain the scientific background and rationale for the investigation being reported*	p. 5	[With 64.3 million … majority of these studies investigated LS7]
Objectives	*3*	*State specific objectives, including any prespecified hypotheses*	p. 5	[Therefore, the aim of…. EPIC-NL cohort]
**Methods**				
Study design	*4*	*Present key elements of study design early in the paper*	p. 6-7, 9-11	Method section paragraphs: Study population, Data collection and preparation, Outcome, Statistical Analysis
Setting	*5*	*Describe the setting, locations, and relevant dates, including periods of recruitment, exposure, follow-up, and data collection*	p. 6-11	Method section
Participants	*6*	*(a) Cohort study—Give the eligibility criteria, and the sources and methods of selection of participants. Describe methods of follow-up*	p. 6, p. 7, p. 9	[EPIC-NL consists of … final sample for analysis of 37,803 people] [Questionnaires were sent … sleep duration] [Participants were followed … with fifth-digit 1 or 3],
		(b) Cohort study—For matched studies, give matching criteria and number of exposed and unexposed	[NA]	[NA]
Variables	*7*	*Clearly define all outcomes, exposures, predictors, potential confounders, and effect modifiers. Give diagnostic criteria, if applicable*	p. 7-11	Method section paragraphs: Life's Essential 8, Life's Simple 7, Outcome, Statistical Analysis

Data sources/ measurement	*8**	*For each variable of interest, give sources of data and details of methods of assessment (measurement). Describe comparability of assessment methods if there is more than one group*	p. 6-9	Method section paragraphs: Data collection and preparation, Life's Essential 8, Life's Simple 7
Bias	*9*	*Describe any efforts to address potential sources of bias*	p. 10-11	[Two multivariable models were created … were used in the categorical analyses] [Sensitivity analyses for … possible reverse-causation bias]
Study size	*10*	*Explain how the study size was arrived at*	p. 6	[EPIC-NL consists of … aged 20-59 years] [We excluded participants … sample for final analysis of 37,803 people], Supplemental figure 1

Supplemental Table 2: Description of the cardiovascular health metrics, how they are measured, and quantified to obtain the Life's Essential 8 score indicatory of cardiovascular health

LE8 Component	Measurement	Score	Definition
Diet	DASH diet adherence	0	1st-24th percentile
		25	25th-49th percentile
		50	50th-74th percentile
		80	75th – 94th percentile
		100	≥95th percentile
Physical activity	Self-reported minutes of activities of at least moderate intensity (MET≥3 (≥55 y) or MET ≥4 (<55 y)) per week and occupational activity	0	0
		20	1-29
		40	30-59
		60	60-89
		80	90-119
		90	120-149
		100	≥150 or (heavy) manual work in occupation
Nicotine exposure	Self-reported use of cigarettes and second-hand smoke exposure	0	Current smoker
		25	Former smoker, quit <1 year
		50	Former smoker, quit 1-<5 years
		75	Former smoker, quit ≥5 years
		100	Never smoker
		-20	Subtract if living with active indoor smoker in home (except when 0)
Sleep health	Self-reported average hours of sleep per night	0	<4
		20	4-<5
		40	5-<6 or ≥10
		70	6-<7
		90	9-<10
		100	7-<9
Body mass index	Body weight divided by height squared (kg/m^2)	0	≥40.0
		15	35.0-39.9
		30	30.0-34.9
		70	25.0-29.9
		100	<25
Blood lipids	Non-HDL cholesterol (mg/dL) calculated from plasma total- and HDL-cholesterol	0	≥220
		20	190-219
		40	160-189
		60	130-159
		100	<130
		-20	Subtract if treated level (except when 0)
Blood glucose	Fasting blood glucose (mg/dL) and/or HbA1c (%)	0	Diabetes with HbA1c ≥10.0
		10	Diabetes with HbA1c 9.0-9.9
		20	Diabetes with HbA1c 8.0-8.9
		30	Diabetes with HbA1c 7.0-7.9
		40	Diabetes with HbA1c <7.0

		60	No diabetes and FBG 100-125 (or HbA1c 5.7-6.4) (prediabetes)
		100	No history of diabetes and FBG < 100 (or HbA1c <5.7)
Blood pressure	Systolic and diastolic blood pressure (mmHg)	0	≥160 or ≥100
		25	140-159 or 90-99
		50	130-139 or 80-89
		75	120-129 / <80
		100	<120 / <80
		-20	Subtract if treated level (except when 0)

Abbreviations: BMI= body mass index; BP=blood pressure; DASH= Dietary approaches to stop hypertension; FBG= fasting blood glucose; HbA1c= haemoglobin A1c; HDL= high-density lipoprotein; PA= physical activity

Supplemental Table 3: Description of the cardiovascular health metrics, how they are measured, and quantified to obtain the Life's Simple 7 score indicatory of cardiovascular health

**LS7 component**	**Score**	**Definition**
Nicotine exposure	0	Current smoker
	1	Former smoker, quit ≤ 12 months ago
	2	Never smoker, or quit > 12 months ago
BMI	0	≥ 30 kg/m^2
	1	25 – 30 kg/m^2
	2	≤ 25 kg/m^2
Physical activity (CPAI) (26)	0	Inactive: Sedentary job and no recreational activity
	1	Moderately inactive or moderately active: sedentary job with <1h recreational activity per day; *or* standing job with <0.5 h of recreational activity per day; *or* physical job with no recreational activity
	2	Active: sedentary job with >1h recreational activity per day; *or* standing job with >0.5h recreational activity per day; *or* physical job with at least some recreational activity per day; *or* heavy manual job
Diet	0	0-1 healthy diet components
	1	2-3 healthy diet components
	2	4-5 healthy diet components
Total cholesterol	0	≥ 240 mg/dL
	1	200-240 mg/dL, or ≤200 mg/dL with treatment
	2	≤ 200 mg/dL
Blood pressure	0	SBP ≥ 140 mmHg or DBP ≥ 90 mmHg
	1	SBP 120-140 mmHg or DBP 80-90 mmHg or treated to < 120/80 mmHg
	2	SBP ≤ 120 mmHg and DBP ≤ 80 mmHg, not treated
Blood glucose	0	≥ 126 mg/dL (fasting), ≥200 mg/dL (non-fasting), diabetes diagnosis with no glucose measurement available
	1	100-125 mg/dL (fasting), 140-200 mg/dL (non-fasting), diabetes diagnosis with treatment and fasting glucose ≤100 mg/dL
	2	≤ 100 mg/dL (fasting), ≤ 140 mg/dL (non-fasting) or no diabetes diagnosis and no or unknown treatment

Abbreviations: BMI = body mass index; CPAI = Cambridge physical activity index; DBP = diastolic blood pressure; LS7 = Life's essential 7; SBP = systolic blood pressure

Supplemental Table 4: Description of the healthy diet components

Healthy diet score components
Fruit and vegetables (≥ 375 grams/day)
Fish (≥ 200 grams/week)
Whole grains (≥ 50 grams/day)
Sodium (< 1500 mg/day)
Sugar sweetened beverages (≤ 450 kcal/week)

Supplemental Table 5: Number of missing for the imputed variables used in the baseline table and/or models

Imputed variable	Missing (N)	Missing (%)
**Demographics**		
Education	234	0.6
Marital status	196	0.5
**Lifestyle factors**		
Smoking	7781	20.6
Current environmental exposure to smoke	946	2.5
Pack year	998	2.6
Number of healthy diet components	179	0.5
DASH adherence	179	0.5
Alcohol use	1243	3.3
Kcal consumption	179	0.5
PA minutes	5052	13.4
Type of occupational PA	5397	14.3
Average hours of sleep	24545	64.9
**Clinical measurements**		
BMI	28	0.1
Waist to hip ratio	72	0.2
Total cholesterol	1516	4.0
HDL cholesterol		4.1
Non-HDL cholesterol	1572	4.2
Systolic BP (mmHg)	88	0.2
Diastolic BP (mmHg)	68	0.2
Pulse (beats/min)	100	0.3
Glucose (mg/dL)	17121	45.3
HbA1c (%)	32556	86.1
**Comorbidities & medication**		
General health	12553	33.2
Prevalence of T2DM	248	0.7
Familial history of T2DM	2202	5.8
Diabetes treatment	21038	55.7
Previous stroke	244	0.6
Prevalent cancer	192	0.5
**LS7 and LE8 components**		
LS7 nicotine exposure	7781	20.6
LS7 BMI	28	0.1
LS7 diet	179	0.5
LS7 cholesterol	1516	4.0
LS7 blood pressure	44	0.1
LS7 blood glucose	330	0.9
LE8 nicotine exposure	8300	22.0
LE8 BMI	28	0.1

LE8 PA	5285	14.0
LE8 diet	179	0.5
LE8 sleep health	24545	64.9
LE8 blood lipids	1572	4.2
LE8 blood glucose	32021	84.7
LE8 blood pressure	50	0.1

Abbreviations: BMI = body mass index; BP = blood pressure; CPAI = Cambridge physical activity index; DASH = dietary approaches to stop hypertension; HbA1c = haemoglobin A1c; HDL = high density lipoprotein; LE8 = life's essential 8; LS7 = life's simple 7; PA = physical activity

Supplemental Table 6: Associations between Life's Essential 8 and risk of heart failure after exclusion of HF cases within 2 years of follow-up

	N_{Events} / N_{total}	*Model 1*	*Model 2*	*Model 3*
**LE8 CVH category**				
Low (0-49)	114 / 1,613	1.00	1.00	1.00
Moderate (50-79)	500 / 28,156	0.28 (0.21-0.38)	0.33 (0.25-0.44)	0.35 (0.26-0.46)
High (80-100)	41 / 7,999	0.10 (0.07-0.14)	0.16 (0.11-0.24)	0.18 (0.12-0.26)

Model 1: crude model; Model 2: adjusted for sex, age, and education; Model 3: adjustments model 2 with addition of marital status, previous acute myocardial infarction, and prevalent atrial fibrillation.

Abbreviations: CVH = cardiovascular health; LE8 = Life's Essential 8; N = number

Values shown are hazard ratio (95% confidence interval).

Supplemental Table 7: Baseline characteristics stratified for CVH reclassification.

LS7 CVH	**Low**	**Low**	**Moderate**	**High**	**Moderate**	**High**
LE8 CVH	**Low**	**Moderate**	**Moderate**	**Moderate**	**High**	**High**
	N = 1604 (4.2%)	*N = 14,510 (38.4%)*	*N = 11,115 (29.4%)*	*N = 2,659 (7.0%)*	*N = 2,300 (6.1%)*	*N = 5,615 (14.9%)*
**Demographics**						
**Age**	53.7 [49.9, 59.2]	53.9 [48.5, 59.5]	50.8 [40.6, 57.2]	46.5 [33.9, 53.6]	51.6 [41.6, 57.5]	44.0 [32.1, 52.5]
**Sex** (female)	73.7	73.0	75.4	77.8	76.7	75.8
**Married**	73.1	74.4	70.9	68.2	67.9	58.9
**Education**						
Low	55.3	44.8	35.8	32.4	29.5	22.3
Middle	38.6	41.0	45.6	47.0	36.9	40.4
High	6	14.2	18.6	20.6	33.6	37.3
**Lifestyle factors**						
**Smoking**						
Current	78.1	55.5	37.3	17.9	16.8	4.1
Never	11.1	35.1	46.0	49.8	75.7	80.1
**Drinking alcohol**						
No, never	8.6	6.9	7.2	10.3	5.5	7.6
No, quit	2.7	1.1	1.0	1.6	0.7	0.9
<1 drink / week	33.8	30.1	28.8	29.9	29.6	29.1
Yes	54.9	61.9	62.9	58.2	64.2	62.4
**DASH-diet score**	20.2 ± 3.8	23.5 ± 4.7	23.6 ± 4.7	22.7 ± 4.3	27.8 ± 4.0	26.3 ± 4.5
**Min of moderate-vigorous PA**	629 [109, 1620]	819 [340, 1726]	728 [343, 1550]	687 [389, 1279]	642 [348, 1508]	619 [372, 1043]
**Sedentary job**	16.0	21.7	22.0	19.9	35.6	31.3
**Average sleep duration** (h)	6.5 ± 1.7	7.1 ± 1.1	7.0 ± 1.2	6.7 ± 1.3	7.4 ± 0.8	7.2 ± 0.9
**Clinical measurements**						
**BMI**	30.9 [28.1, 33.9]	26.8 [24.8, 29.5]	24.8 [22.8, 27.0]	23.6 [22.0, 25.0]	23.9 [22.1, 25.7]	22.9 [21.5, 24.2]
**Systolic BP**	145 ± 21	134 ± 19	124 ± 17	116 ± 13	121 ± 15	113 ± 11
**Diastolic BP**	88 ± 11	82 ± 10	77 ± 10	73 ± 8	75 ± 9	71 ± 8

**Total cholesterol**	249 ± 41	233 ± 39	210 ± 38	193 ± 31	205 ± 37	183 ± 31
**HDL cholesterol**	47 ± 13	54 ± 16	57 ± 16	56 ± 14	65 ± 18	61 ± 16
**Blood glucose**	101 [92, 123]	94 [86, 104]	90 [81, 99]	88 [81, 95]	88 [79, 97]	86 [78, 94]
**Life's Essential 8 total score and metrics**						
**LE8 score**	44.4 ± 4.5	62.5 ± 6.9	70.9 ± 5.4	74.8 ± 3.7	83.0 ± 3.1	86.4 ± 4.6
**Sleep health**	76.3 ± 18.9	85.0 ± 14.2	85.1 ± 15.5	82.9 ± 17.7	95.3 ± 11.6	90.4 ± 12.2
**BMI**	42.5 ± 26.1	68.6 ± 25.6	83.1 ± 20.6	91.9 ± 14.5	89.4 ± 15.4	96.1 ± 10.4
**BP**	32.3 ± 23.4	52.3 ± 27.4	68.4 ± 28.6	81.1 ± 25.3	76.5 ± 24.2	90.3 ± 18.5
**Diet**	16.5 ± 22.3	40.1 ± 31.9	40.2 ± 31.5	32.7 ± 29.4	70.1 ± 25.8	59.2 ± 29.9
**Blood glucose**	61.8 ± 27.4	81.1 ± 22.9	88.0 ± 18.9	91.0 ± 17.0	93.9 ± 14.5	95.8 ± 12.4
**Blood lipids**	24.2 ± 22.0	39.7 ± 27.3	57.8 ± 28.9	69.6 ± 25.3	70.6 ± 28.2	83.9 ± 23.2
**PA**	84.4 ± 29.5	96.3 ± 14.0	96.9 ± 12.3	96.5 ± 13.3	99.1 ± 5.1	99.0 ± 5.4
**Nicotine exposure**	16.0 ± 29.9	38.1 ± 42.1	50.3 ± 40.8	56.5 ± 34.2	75.6 ± 35.3	80.8 ± 25.5
**Life's Simple 7 total score and ideal metrics**						
**LS7 score**	5.0 ± 1.4	7.1 ± 1.1	9.4 ± 0.5	11.2 ± 0.5	9.8 ± 0.4	11.7 ± 0.7
**Ideal BMI**	5.5	26.8	53.0	74.9	66.3	87.3
**Ideal BP**	2.7	15.3	37.6	60.5	43.7	75.7
**Ideal blood glucose**	77.3	93.1	98.9	99.7	98.4	99.7
**Ideal blood lipids**	8.1	15.4	40.1	67.5	40.0	73.5
**Ideal diet**	1.1	1.2	2.7	5.7	1.1	4.6
**Ideal PA**	25.7	25.8	50.5	76.7	22.3	60.4
**Ideal nicotine exposure**	20.8	42.0	59.9	77.1	78.9	92.1

Values are shown in percentages, mean ± standard deviation, or median [interquartile range]. LS7 and LE8 scores and components are the calculated averages of 10 imputed datasets. *Abbreviations:* BMI = body mass index; BP = blood pressure; CVH = cardiovascular health; DASH = dietary approaches to stop hypertension; HDL = high density lipoprotein; LE8 = Life's essential 8; LS7 = Life's simple 7; PA = physical activity.
